# Correction: Is social cohesion produced by weak ties or by multiplex ties? Rival hypotheses regarding leader networks in urban community settings

**DOI:** 10.1371/journal.pone.0307185

**Published:** 2024-07-10

**Authors:** Silvio Salej Higgins, Neylson Crepalde, Ivan L. Fernandes

Figs 1 and 2 are uploaded incorrectly. Please see the correct Figs 1 and 2 here.

**Fig 1:**
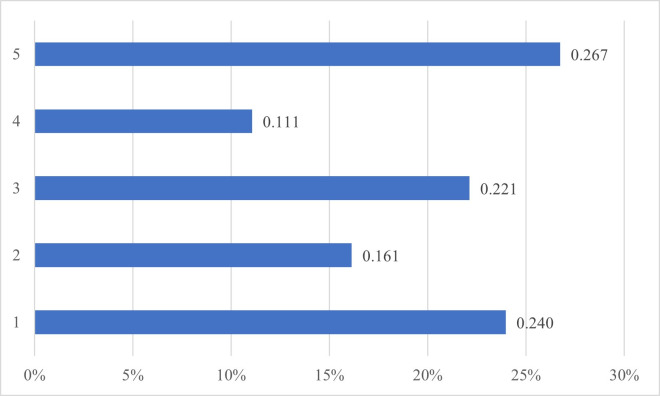
Alpha community strength of ties by frequency.

**Fig 2:**
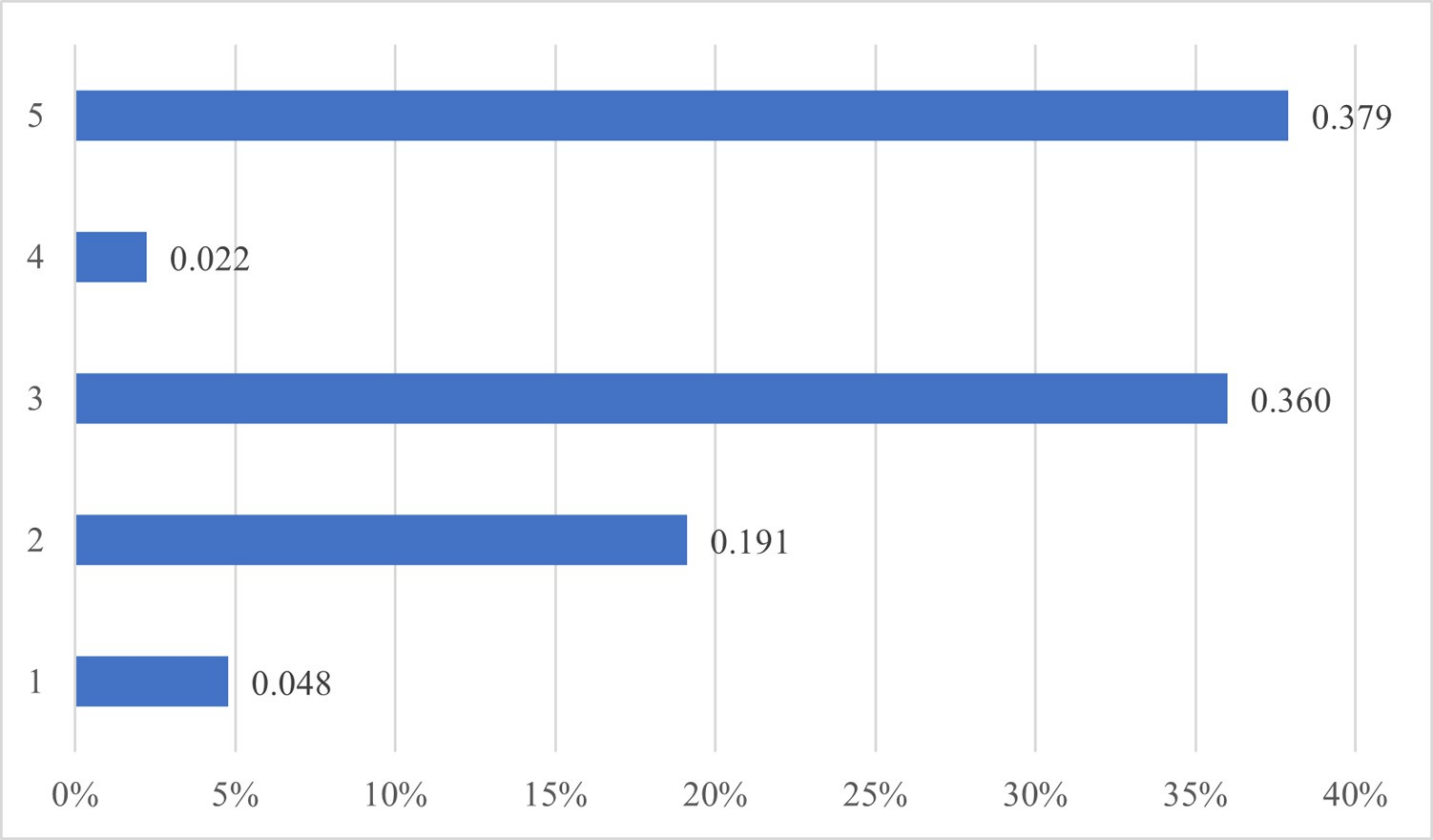
Beta community strength of ties by frequency.
