# Supplementary material for: Is social cohesion produced by weak ties or by multiplex ties? Rival hypotheses regarding leader networks in urban community settings
Source: PLoS One. 2021 Sep 27;16(9):e0257527. doi: 10.1371/journal.pone.0257527 (PMC8475979; doi:10.1371/journal.pone.0257527)
Supplement: S1 File — (DOCX) [file pone.0257527.s002.docx]

**Is social cohesion made by weak ties or multiplex ties?**

**Rival hypotheses regarding leader networks in urban community settings**

**Silvio S. Higgins^a^** and **Ivan L. Fernandes^b^**

^a^Associated Professor of Sociology, Federal University of Minas Gerais (Brazil)

^b^Research Assistant, Federal University of Minas Gerais (Brazil)

**Abstract**

In his seminal work, The strength of weak ties (1971), Mark Granovetter challenged sociologists to test sociometric hypothesis regarding collective action in communitarian settings. In this article, we have tested the two main hypotheses which consider social cohesion in communitarian urban settings: the cohesion by weak ties versus cohesion by multiplex ties. We have studied the elite leaders in two slum communities of Belo Horizonte (Brazil). Three social processes were examined as multiplex interactions: recognized status, exchange of useful information and collaboration. Our findings reveal, on the one hand, that multiplexity is a function of the strength of ties and, on the other, that reciprocity and shared domains of performance fuel such multiplexity. If we assume that high orders of leaders are global cohesive social circles, that is, a network of social hubs, our findings confirm cohesion by multiplexity. Multiplex ties, analyzed in entangled and disentangled ways, are strong ties determined by reciprocity, high frequency of interactions and shared domains of social action.

**Key words:** weak ties, strong ties, social cohesion, collective action and multiplexity.

**Introduction**

At the end of his seminal article *The Strength of Weak Ties* (1971), Mark Granovetter encouraged the sociological community to test new hypotheses concerning how weak ties function in order to improve collective action. When considering the urban public policies launched in Boston in the 1950s, he revealed a set of new insights on the social cohesion of urban segregated communities: (a) a community without rich networks of weak ties would not be capable of collective action; (b) if organizational life and workplace are the sources of weak ties, then bedroom neighborhoods would not be able to create bridge ties beyond the immediate inner circles of people; (c) the lack of weak ties affects confidence between leaderships and the grassroots. From Granovetter’s perspective, the aforementioned conjectures would be useful for understanding the critical case of the West End - an Italian community in Boston: The question is why this community was unable to address a public top-down project that dramatically affected local life?

This question, grounded in the former set of conjectures, merited a reply *in extenso* from Herbert Gans (1974). The *American Journal of Sociology* understood the importance of the problem and published this exciting sociological debate, which was somewhat unusual in the sociological setting (Gangs, 1974; Granovetter, 1974). From Gans’ point of view, only middle-class communities would be able to create a cohesive confidence between leaders and the grassroots. Granovetter asked why this was. Despite his recognition of heuristic power in the weak ties hypothesis, Gans (1974) highlights some of Granovetter’s misunderstandings of the social context of the West End Community. Firstly, there were weak ties but the community was also fragmented in space. Secondly, weak ties depend on history and cultural factors. In the West End, there was no tradition of social struggles seeking to improve the welfare of the community. Anyone who opposed the project would be disapproved of by their peers because the Catholic Church supported the Government’s urbanistic intervention in local life. Finally, the only leader working in the neighborhood was a white man who was mistrusted by black people. As a balanced conclusion, Granovetter (1974) accepted that weak ties were both the cause and consequence of history and culture.

Granovetter was convinced of the theoretical power of his framework in terms of prediction of collective action, taking into account the extent to which weak ties bridge clustered communities, albeit recognizing the methodological limits of his challenge:

“In the absence of actual network data, all this is speculation. The hard information needed to show either that the West End was fragmented or that communities which organized successfully were not, and that both patterns were due to the strategic role of weak ties, is not at hand and would not have been simple to collect. Nor has comparable information been collected in any context” (Granovetter, 1973, p. 1375)

**Later studies on community networks**

After its original formulation, the hypothesis of weak links has been thoroughly examined by social scientists. Susan Greembaum (1982) developed a state of the art treatment on the mechanisms of cohesion in urban communities. Beyond the debate between Gans and Granovetter, and seeking evidence to test the cohesive strength of weak bonds, she invokes Wellman's (1979) findings on the role of intimate relationships in sparse networks beyond the local community. Based on a survey applied to four urban communities of worker strata in Kansas, Greenbaum proposes an alternative hypothesis to that formulated by Granovetter: bridge ties, vital for the general cohesion of an urban community, consist of multiplex type interactions, where family relationships can be superimposed onto co-participation in community organizations, besides being endowed with affective intensity. That is, strong, multiplex type ties can be bridge ties.

Their results indicated that the communities studied were made up of interaction networks linked to spatial proximity. In the first place, there were intra block-face networks formed of neighbors of residential units close to each other. In this type of cluster, the main ties between acquaintances were latent relationships that fit the concept of weak ties proposed by Granovetter. Secondly, there were inter block-face networks, composed of multiplexed loops where relationships of kinship, belonging to the same work, club or church, old childhood friendships, etc. were superimposed. In summary, strong-multiplex ties, between spatially sparse neighbors, can be understood as a source of greater cohesion between social clusters, facilitating effective communication on a broad geographical scale and a greater sense of identity beyond face-blocks.

In summary, Greenbaum found two pieces of evidence contrary to Granovettter's hypothesis:

- Weak ties structure clusters spatially close.

- Strong multiplex ties bridge spatially dispersed clusters.

Many years later, Robert Sampson (1997, 2002) developed a pioneering study that corresponded to the two elements of Granovetter’s puzzle: the theoretical role of weak ties to bridge clustered communities and the methodological strategy for collecting network data. In *Leadership and the higher-order structure of elite connections* (Sampson, 2002), the author proposed a strategy for understanding the covariance between the collective efficacy of communities and the network structure of the elite leaders in the city of Chicago. In order to understand the endogenous capability for collective action in a community, Sampson built an index entitled *Collective Efficacy,* integrating attitudinal and objective factors. Survey data are the informational basis of the index. For example, basic survey questions aim to discover whether neighbors react to observed misconducts such as drug use or antisocial actions against public amenities.

In Sampson’s research strategy there are several elements that need to be highlighted in terms of how he addresses Granovetter’s puzzle. Firstly, the focus was on what Sampson called “high order structure”. This cannot be confused with high order parameters in exponential random graph models (Lusher et al.2013). The former corresponds to an interactional order among selected people, whereas the latter is a supra-triadic isomorphism in the formal analysis of the theory of graphs. In the empirical field of Chicago City, what was considered high order was the elite leaders identified by a mixed strategy that included secondary data and snowball sampling. In this sense, there was a double problem to be solved. The first of these was how to identify those who were considered people who “get things done” in the community. The second was what the boundary of that social universe called “elite” was which would be targeted by the researcher. Sampson’s insight stated that a network of key leadership groups creates systematic and influential connections both within and among communities.

Secondly, the sampling plan identified six different realms where a citizen could be considered a leader: education, politics, religion, business, law enforcement, and community organization. Forty-seven community zones were selected from all social strata from the 77 administrative zones into which the city was divided.

Thirdly, a *geocoded list* was created with 10,000 names of individuals identified as leaders. The data were collected from diverse sources such as telephone books and business and service directories. Fourthly, around 5,500 leaders were identified in the 47 areas, and attributes of personal identity, work and location were collected. Fifthly, 2,500 cases were selected and stratified by community and realms. Sixthly, more than 1,700 interviews were conducted among the selected cases. Lastly, a snowball sampling was performed in order to generate new names, asking who the most influential people in the six realms were and beyond that. In the end, more than 3,800 new names were generated.

The former sampling process was repeated as a panel survey seven years later, in 2002. This second round aimed to investigate: (a) the permanence of leaders; (b) the emergence of new leaders in old positions or in new organizations; (c) the trajectory of leaders. Due to a decreased budget, only 30 communities out of the former 77 were included in the new sample. A high turnover was found because only 60 percent of respondents were in positions similar to those of the first round.

This overview of Sampson’s strategy enables us to better understand the two challenges posed by Granovetter. When he said that there was no available data to test the weak ties hypothesis, he was referring to a major problem in social network analysis: How to sample a structured set of interactions? This is not a trivial problem because structural research runs opposite to standard survey sampling. Intrinsically, a network is a structure. Thus, the researcher must assume that its elements are interdependent. In contrast, standard survey sampling works on the assumption of independent observations. Therefore, the challenge consists in preventing one observation from introducing selection bias into others. This is the elementary heritage of the positivistic point of view that constrains subjective bias when the researcher is observing the social world (Durkheim, 2014[1885]; Babbie, 2001).

At this point, some limits in Sampson’s strategy must be highlighted. The elite network is not a representative sample in the standard meaning of survey research. The universe of leaders and their interactions is a snip made by the observer. As with any social network research, when defining the boundaries of the object, the researcher considers some plausible criteria such as the seven steps mentioned before. Consequently, we know nothing about the structural relations between the elite and grassroots, which was one of the key puzzling problems presented by Granovetter. Nevertheless, given the state of art nature of network sampling, we could not hide this tradeoff between boundary and representation (Stivala et al., 2016).

**Rival hypotheses**

Put in black and white, the seminal and broad hypothesis proposed by Granovetter (1973), affirms that weak ties operate as bridging and cohesive links in spatially sparse settings, which we refer to as *globalcohesion by weak ties*. On the one hand, the rival and narrow hypothesis, as stated by Greenbaum (1982), argues that multiplex ties operate as bridging and cohesive links in spatially sparse settings, which we refer to as *global cohesion by multiplex ties*. On the other hand, weak ties work as bridging and cohesive links in spatially close settings, which we refer to as *local cohesion by weak ties*. Table 1 depicts the set of aforementioned theoretical hypotheses. With these options available, it would be possible to model a set of data under a triple condition: having multiplex data, spatial information, and data about the strength of ties. In the following, we reveal how we were able to address the challenges of these rival hypotheses.

**Table 1 – Network data and hypothesis on social cohesion**

|  | **Multiplexity** | **Strength of ties** |
| --- | --- | --- |
| **Global space** | *Global cohesion by multiplex* ties (Greenbaum, 1982) | *Global cohesion by weak ties*  (Granovetter, 1973) |
| **Local space** |  | *Local cohesion by weak ties*  (Greenbaum, 1982) |

**Our object: two multiplex systems of social status in impoverished urban communities**

Addressing Grannovetter’s puzzle and inspired by previous studies, two impoverished urban communities in Belo Horizonte (Brazil) were selected, seeking to better understand social exchange and interdependencies between the elite of their leaderships. Methodologically, we have gone further than Greenbaum (1982) and Sampson (1997, 2002). In contrast to the former, who worked with ego-networks via survey, we collected a complete set of multiplex networks. The advantage of working with a well-defined boundary where each one of respondents could indicate all their *alteri* is well known (Krackhardt, 1987). Furthermore, we have taken into consideration the multiplex nature of interactions between leaderships. That is, an interaction process, between two or more actors, which is simultaneously a flux of different resources or an interpretive exchange of random meanings. At the beginning of sociological work, Simmel (1989) stressed how socialization was a multiple and simultaneous process across social circles, such as family, school, friendship, work, leisure etc. Recently, in a technical sense, White (1976, 2002, & 2008) has developed tools for understanding the social structure from multiple networks.

In our study, we collected information on five different social processes among leaderships of two urban communities: perceived status, exchange of useful information, coordination/collaboration, lateral control, conflicts and bridging social capital. The quality of answers was not equal across the five sociometric generators. Questions on lateral control and conflict faced resistance and evasive attitudes from the respondents. Therefore, the rate of answers was not high. The answers to the final name generator, regarding bridging social capital, demanded additional treatment in order to create categories of professional fields from where some external resources arrive at the communities. Consequently, the data revealed a basic multilevel modeling. For these reasons, we have restricted the analysis here to the first three sociometric generators (perceived status, exchange of useful information, and coordination/collaboration) whose answers could be modeled with the same statistical tool: Exponential Random Graph Model (ERGM). Finally, and in contrast to both Greenbaum and Sampson, in the first sociometric generator we collected a proxy concerning the strength of ties, using an interaction frequency scale.

Both communities have a similar profile of income, infrastructure, and public services. However, there was a marked difference in terms of their history. The Alpha community, in contrast to the Beta community, had a long history of violence and homicides related to drug trafficking, as reported in the official database of the police . This is useful background information that permits a better interpretation of the relational dynamic among leaders.

***Data collection***

Seeking to identify the elite leaders, snowball sampling was conducted in both communities using two protocols. The first protocol differentiated eight key community realms where endogenous leaders operated: education, religion, politics, security, health, business, local organization, sports, and leisure. As a first step, a seed of leaders’ names was generated by interviewing key informants in hospitals, schools, grocery stores, associations, churches and so on for each realm. Each respondent mentioned five names of people perceived as influential in the community. As a second step, a new round of interviews was conducted to generate another five names. The process ended when we were unable to find new indications, that is, by saturation. The second protocol, with the complete roster of leaders in each community, formulated six sociometric questions on different social processes that were relevant in the exchange and local recognition between leaders. The following were the main processes studied with the respective sociometric questions:

- Perceived status betweeen leaders,

*Thinking about last year. Of the people on this list, who do you consider to be leaders that are working in a helpful way for the benefit of the community? You can choose up to ten names.*

*How often have you spoken with each of them?*

*Weekly*

*Fortnightly*

*Monthly*

*Bi-annually*

*Annually*

- Exchange of useful information

*Thinking about last year. Of the people on this list, who have you turned to requesting any kind of useful information for your work with the community? You can choose up to ten names. Why have you chosen each one of them?*

- Coordination/Collaboration

*Thinking about last year. Of the people on this list, who have you contacted to organize any activity for your community (such as improvements in the school, cleaning the square, helping in the vaccination campaign, etc.). You can choose up to ten names.*

Two sets of data were collected in each community: firstly, a multiplex network, that is, different interactional structures between the same agents; secondly, relevant attributes on nodes and relationships. The following table summarizes the data collected:

**Table 2 – Data collected**

| **Multiplex Networks** | **Attributes on nodes and relationships** |
| --- | --- |
| - Status - Information - Coordination/Collaboration | - Realms of leadership - Strength of ties |

The scale of frequency in the first sociometric generator enabled the construction of a proxy variable for the strength of ties. Despite the fact that we do not have any information regarding emotional support between leaders, which is a key element in the canonical definition of strength (Granovetter, 1973), we consider the frequency of interactions as a necessary condition of the strength of ties in the context of community activity. The leaders, identified endogenously by key informants in the neighborhood, shared a social space without a specific division of labor. Mutual and emotional support is not intrinsic to these leaders’ voluntary activities. The original scale was dichotomized using the weekly frequency as a cut-off point for defining a strong tie network and all the other frequencies were considered weak ties.

Once again, we must remember that in this research we did not collect data on how strong or weak the relations between the perceived leaders and grassroots are. A survey sampling strategy aimed to track the interactions between the elite and the neighborhood would pose significant and almost intractable problems with missing data (Butts, 2010, Koskinen et. al. 2013). We identified a perceived elite of leaders and then relevant data about exchanges between them were collected. With previous warnings in mind, and taking into account Granovetter’s seminal puzzle, we can test the rival hypotheses about social cohesion.

**Descriptive results**

Some descriptive statistics enable the comparison of both groups of elite leaders as regards the level of cohesion in each of the networks. The Alpha Community has an elite of 32 leaders and the Beta Community has 40. Only three social processes - perceived status, search for useful information and collaboration - were included in this analysis, due to the accuracy and completeness of data for both communities. We prepared three different square matrices with the respective number of lines and columns.

**Table 3 - Cohesive metrics**

|  | **Density** | | **Avg Distance** | | **Compactness index** | | **Diameter** | |
| --- | --- | --- | --- | --- | --- | --- | --- | --- |
|  | Alpha | Beta | Alpha | Beta | Alpha | Beta | Alpha | Beta |
| **Status*** | 0.219 | 0.202 | 1.924 | 2.124 | 0.508 | 0.494 | 4 | 5 |
| **Information exchange** | 0.104 | 0.114 | 2.666 | 2.424 | 0.328 | 0.302 | 7 | 6 |
| **Collaboration** | 0.089 | 0.143 | 3.004 | 2.567 | 0.309 | 0.399 | 7 | 7 |

*Binarized by the minimum value in the scale

Table 2 depicts, in a comparative way, how cohesive the three different processes studied are. In both communities, the status system reveals a denser recognition exchange. In terms of information exchange and collaboration, the Alpha community presents a slightly weaker density if compared with the Beta community. The intrinsic characteristics of each social process could explain this difference. For example, being recognized or recognizing others as leaders does not depend on actual collaborative relationships.

When comparing the frequencies of interactions (Figures 1 and 2), in the Alpha Community, 60 percent of the ties show a monthly or higher frequency, whereas in the Beta Community this performance is 76.11 percent – scale: 1=annually, 2=bi-annually, 3=monthly, 4=fortnightly and 5=weekly - . This could suggest a closer status interactive system in the Beta Community. Two covariate matrices were constructed from this attribute: one for strong ties, using the weekly frequency as the cut-off point, and the other for the weak ties, using the yearly frequency.

**[insert Digraph 1.]**

In order to explore the exchanges between status realms, we collapsed the actors’ nodes. In the Alpha Community (Digraph 1), there is an intensive social circuit between leaders operating in the areas of education, safety, health, sports, culture, and leisure. This digraph is a useful tool for understanding what realms are crucial for community life. In the Beta Community (Digraph 2), the main social circuit includes some realms that were not important in the previous one, such as local organization, business, or protestant religion. In this case, we distinguished the Catholic priest from Pentecostal pastors. Health and safety were peripheral in this case.

**[insert Digraph 2.]**

In terms of multiplexity, the two digraphs represent interactions between leaders encompassing the three social processes: status, exchange of useful information and collaboration. An option for the strict sense of multiplex ties was included. As can be seen, some leaders became isolated after having been cut out of the single and double interactions. In a substantive way, the resultant digraphs can be interpreted as the cohesive core of both social systems.

**[insert Digraph 3.]**

**[insert Digraph 4.]**

**Exponential Random Graph Models (ERGM) to test hypotheses on cohesion**

Taking into account that we only have data on two out of four conditions supposed by the rivalry between Granovetter and Greenbaum, we posed the following questions that permit the contrast of our evidence with the previous results of sociological literature.

**Table 4 –Some new questions on local cohesion**

|  | **Strong ties**  **High frequency** | **Weak ties**  **Low frequency** |
| --- | --- | --- |
| **Multiplexity** | *Are strong ties determinant for the creation of multiplex ties between leaders?* | *Are weak ties determinant for the creation of multiplex ties between leaders?* |
| **Non multiplex or single ties** | *Are strong ties determinant for status, exchange of useful information and collaboration taken separately*? | *Are weak ties determinant for status, exchange of useful information and collaboration taken separately*? |

In an attempt to answer the former questions, we separately modeled the multiplexity and the three processes one by one. The idea is to understand what factors, whether endogenous or exogenous, determine the emergence of both kinds of networks (multiplex or single) between social leaders. Thus, we collected the data as a cross-sectional study which was not present in Granovetter’s or Greenbaum’s work. We looked for the existence of any relation between the strength of ties and multiplexity.

Using exponential random graph models (ERGMs), we proceeded in two steps. Firstly, we modeled the social system as a multiplex process. In other words, the interactions composed by the three processes were considered simultaneously. Secondly, we treated every social system as an independent process, step by step, questioning if the strength of ties makes any difference in the intrinsic nature of interactions.

The general form of the model is the following:

$\Pr\left( X=x | \theta\right)≣ P_{\theta}(x)=\frac{1}{k\left( \theta\right)}exp\{\theta_{1}z_{1}\left( x \right)+ \theta_{2}z_{2}\left( x \right)+\ldots\theta_{p}z_{p}\left( x \right)\}$(1)

The probability of each parameter is:

$\Pr\left( X_{ij}=1 | \theta\right)= \frac{\exp\theta}{1+\exp\theta}$(2)

***Results on multiplexity***

ERGMs are useful for understanding the endogenous social process that produces an interactional social system. The search focus are the determinants, whether endogenous or the result of interactions – dyadic, triadic and supra-triadic – be exogenous or the result of actors and settings, that could explain the emergence of a social formation (White, 2008; Lusher et. al. 2013). Tables 5 and 6 depict the consequences of multiplexity for both communities. Each model includes three kinds of effects; two endogenous – markovian and dyadic covariate- and one exogenous – categorical attribute. The isolate effect is present because some nodes do not have multiplex links in the resultant graph. We must remember that we have considered only strict multiplexity, that is, the links that encompass the processes at the same time. In a substantive way, the results are:

**Table 5– Multiplexity in the Alpha Community (MPnet)**

| **Model A** | **Effects** | **Parameter** | **Stderr** | **t-ratio** | **Model B** | **Effects** | **Parameter** | **Stderr** | **t-ratio** |
| --- | --- | --- | --- | --- | --- | --- | --- | --- | --- |
|  | ***Markovian*** | | | |  | ***Markovian*** | | | |
|  | **ArcA** | -5.4121 | 0.717 | -0.026* |  | **ArcA** | -5.1828 | 0.714 | 0.018* |
|  | **ReciprocityA** | 2.9172 | 0.657 | -0.082* |  | **ReciprocityA** | 2.7804 | 0.663 | 0.003* |
|  | **IsolateA** | -0.4751 | 0.795 | -0.001 |  | **IsolateA** | -0.4668 | 0.785 | -0.048 |
|  | **AinSA** | 0.0702 | 0.436 | -0.013 |  | **AinSA** | 0.0084 | 0.363 | 0.038 |
|  | **AoutSA** | 1.3357 | 0.881 | -0.026 |  | **AoutSA** | 1.3353 | 0.853 | 0.017 |
|  | ***Social circuit*** | | | |  | ***Social circuit*** | | | |
|  | **A2PA-D** | -0.4170 | 0.470 | -0.034 |  | **A2PA-D** | -0.4483 | 0.451 | 0.008 |
|  | **A2PA-U** | 0.1379 | 0.109 | -0.003 |  | **A2PA-U** | 0.040 | -0.045 | 0.849* |
|  | ***Categorical attribute*** | | | |  | ***Categorical attribute*** | | | |
|  | **Realms_MatchA** | 1.2507 | 0.412 | 0.017* |  | **Realms_MatchA** | 1.3410 | 0.391 | 0.063* |
|  | **Realms Match Reciprocity** | -2.7770 | 1.317 | -0.051* |  | **Realms Match Reciprocity** | -2.9839 | 1.321 | -0.053* |
|  | ***Dyadic covariate*** | | | |  | ***Dyadic covariate*** | | | |
|  | **Low frequency**  **Weak ties** | 1.9127 | 0.310 | 0.003* |  | **High frequency**  **Strong ties** | 2.4477 | 0.361 | 0.080* |

*Significant by Wald’s test

**Table 6– Multiplexity in the Beta Community (MPnet)**

| **Model A** | **Effects** | **Parameter** | **Stderr** | **t-ratio** | **Model B** | **Effects** | **Parameter** | **Stderr** | **t-ratio** |
| --- | --- | --- | --- | --- | --- | --- | --- | --- | --- |
|  | ***Markovian*** | | | |  | ***Markovian*** | | | |
|  | **ArcA** | -4.0207 | 0.641 | 0.039* |  | **ArcA** | -4.4159 | 0.677 | 0.009* |
|  | **ReciprocityA** | 0.8293 | 0.859 | -0.009 |  | **ReciprocityA** | 0.4309 | 0.799 | 0.021 |
|  | **IsolateA** | 1.4226 | 0.661 | -0.038* |  | **IsolateA** | 1.6152 | 0.737 | -0.027* |
|  | **AinSA** | 0.0006 | 0.351 | 0.041 |  | **AinSA** | -0.2374 | 0.396 | 0.022 |
|  | **AoutSA** | 0.6709 | 0.288 | 0.058* |  | **AoutSA** | 0.4005 | 0.321 | 0.005 |
|  | ***Social circuit*** | | | |  | ***Social circuit*** | | | |
|  | **ATA-C** | 0.0688 | 0.267 | 0.066 |  | **A2PA-TDU** | 0.0334 | 0.089 | 0.026 |
|  | **ATA-D** | 0.0213 | 0.413 | 0.099 |  |  |  |  |  |
|  | ***Categorical attribute*** | | | |  | ***Categorical attribute*** | | | |
|  | **Realms_MatchA** | 0.8062 | 0.338 | 0.062* |  | **Realms_MatchA** | 0.9616 | 0.383 | 0.030* |
|  | **Realms Match Reciprocity** | -5.3474 | 16.033 | -0.063 |  | **Realms Match Reciprocity** | -4.5565 | 22.624 | -0.045 |
|  | ***Dyadic covariate*** | | | |  | ***Dyadic covariate*** | | | |
|  | **Low frequency**  **Weak ties** | 0.7057 | 0.254 | 0.004* |  | **High frequency**  **Strong ties** | 3.1819 | 0.299 | 0.030* |

*Significant by Wald’s test

- In the Alpha Community, as we can see in Table 5, the multiplex ties emerge from reciprocal choices between leaders and within the same realms where the leaders are working. That is, reciprocity and the fact of working in the same field (health, education, religion etc.) increase the probability of a multiplex tie between leaders being formed. The frequency of interactions, taken by us as a proxy of strength, reveals a difference between weak ties and strong ties. Strong ties, measured as a weekly frequency, increase the probability of forming multiplex links by 92%, while weak ties, measured as fortnightly to annual frequency, increase a little less, by 87%. In this way, we can reflect that the multiplexity is conditioned by the frequency of ties.
- In the Beta Community, as we can see in Table 6, the results are subtly different. In this case, reciprocity does not have a significant effect on creating multiplex ties, but the same realms are determinants of multiplexity. Isolates are a significant effect in this multiplex system, which is something that does not happen in the Alpha Community. This means that multiplexity creates a boundary that leaves several actors out of the exchange system. The strength of ties reveals an important difference when compared with the Alpha Community. A high frequency of interactions, that is weekly, increases the probability of a multiplex link to be created by 96%, while lower frequencies increase only by 67%. In this case, the effect of the high frequency in the creation of multiplex links is much clearer.

Based on Granovetter’s classical definition of the strength ties – a function of frequency, reciprocity and intimacy, we have observed, in the Alpha Community, how reciprocity controls the difference in frequency, rendering the effect of weekly interactions closer to all the rest. In contrast, in the Beta Community, when reciprocity is not a significant effect, the high frequency of interactions increases the probability of creating multiplex ties if compared with low frequency by 30%.

***Results on three processes considered separately***

As was said before, the results will be presented separately. Here, we analyze social cohesion separately considering the three processes observed.

***Status***

In both communities there was no evidence that could demonstrate the strength of ties, high or low frequency, as a relevant factor in status recognition. Being recognized as an influential leader does not depend at all on the frequency of relationships. Each model, Tables 7 and 8, reveals different effects, or endogenous determinants, that could explain the emergence of both social systems. It must be stressed that reciprocity is a significant effect. In the Alpha Community, to be recognized as a leader is a result of reciprocal choices. In the Beta Community, the result is a little different - to be recognized as a leader is a reciprocal choice restricted to the same realm of performance. For instance, those who work towards improving the educational services in the community are akin to those peers who are in same realm.

**Table 7- Status in the Alpha Community**

| **Model A** | **Effects** | **Parameter** | **Stderr** | **t-ratio** |  | **Effects** | **Parameter** | **Stderr** | **t-ratio** |
| --- | --- | --- | --- | --- | --- | --- | --- | --- | --- |
|  | **Markovian** | | | |  | **Markovian** | | | |
|  | **ArcA** | -4.0205 | 0.348 | 0.054* | **Model B** | **ArcA** | -2.9615 | 0.250 | 0.019* |
|  | **ReciprocityA** | 1.1107 | 0.402 | 0.037* |  | **ReciprocityA** | 0.7739 | 0.308 | -0.031* |
|  | **Transitive-TriadA** | 0.1142 | 0.060 | -0.013 |  | **In3StarA** | 0.1447 | 0.038 | 0.042* |
|  | **Cyclic-TriadA** | -0.1739 | 0.112 | -0.017 |  | **AinAoutSA** | 0.2476 | 0.089 | -0.011* |
|  | **Social circuit** | | | |  | **Social circuit** | | | |
|  | **ATA-T** | 0.2558 | 0.208 | 0.020 |  | **A2PA-D** | 0.3025 | 0.161 | 0.028 |
|  | **Categorical attribute** | | | |  | **Categorical attribute** | | | |
|  | **Realms_MatchA** | 1.2038 | 0.427 | 0.026* |  | **Realms_MatchA** | 0.7793 | 0.307 | 0.098* |
|  | **Dyadic covariate** | | | |  | **Dyadic covariate** | | | |
|  | **Realms_MatchRec** | -0.5409 | 0.722 | 0.043 |  | **Realms_MatchRec** | -0.3058 | 0.642 | 0.006 |
|  | **Low frequency**  **Weak ties** | 13.0289 | 16.000 | 0.063 |  | **High frequency**  **Strong ties** | 10.4268 | 13.022 | 0.078 |

*Significant by Wald’s test

**Table 8 - Status in the Beta Community**

| **Model A** | **Effects** | **Parameter** | **Stderr** | **t-ratio** |  | **Effects** | **Parameter** | **Stderr** | **t-ratio** |
| --- | --- | --- | --- | --- | --- | --- | --- | --- | --- |
|  | **Markovian** | | | |  | **Markovian** | | | |
|  | **ArcA** | 2.3778 | 0.578 | 0.049* | **Model B** | **ArcA** | -8.4981 | 1.116 | -0.060* |
|  | **ReciprocityA** | -0.0648 | 0.315 | 0.020 |  | **ReciprocityA** | 0.5431 | 0.285 | 0.008 |
|  | **OutStarA** | -0.0810 | 0.039 | 0.051* |  | **AinSA** | 0.498 | -0.063 | 0.292* |
|  | **TwopathA** | -0.0208 | 0.030 | 0.048 |  | **AoutSA** | 1.0434 | 0.262 | -0.049* |
|  | **Transitive-Triad** | 0.1392 | 0.063 | 0.052* |  |  |  |  |  |
|  | **Cyclic-Triad** | -0.3369 | 0.120 | 0.024* |  |  |  |  |  |
|  | **Social circuit** | | | |  | **Social circuit** | | | |
|  | **ATA-TDU** | 0.1285 | 0.066 | 0.047 |  | **ATA-C** | -0.0905 | 0.049 | -0.046 |
|  | **Categorical attribute** | | | |  | **Categorical attribute** | | | |
|  | **Realms_MatchA** | -0.5953 | 0.312 | 0.034 |  | **Realms_MatchA** | -0.5805 | 0.407 | 0.007 |
|  | **Realms_MatchRec** | 2.0480 | 0.603 | -0.009* |  | **Realms_MatchRec** | 1.6523 | 0.687 | 0.035* |
|  | **Dyadic covariate** | | | |  | **Dyadic covariate** | | | |
|  | **High frequency**  **Strong ties** | 13.0289 | 16.000 | 0.063 |  | **Low frequency**  **Weak ties** | 12.9364 | 15.972 | 0.063 |

*Significant by Wald’s test

***Exchange of useful information***

This specific process reveals a clear difference between low and high frequencies as a condition that increases the probability of searching for useful information. *Ceteris paribus,* in the Alpha Community, weekly, reciprocal and in the same realm interactions increase the probability of searching for useful information by 94% (Table 9, Model A). When interactions are less frequent, fortnightly or under, reciprocal and in the same realm, the probability of exchanging useful information is only 82% (Table 9, Model B). In the Beta Community, the situation is similar, albeit with a subtle difference. Weekly interactions and reciprocal in the same realm increase the probability of searching for useful information by 86.6% (Table 10, Model A). A fortnightly frequency, or less, increases the probability of creating a new link through which useful information flows by 73.7%. Considering all the other effects, it is important to stress that the Beta Community is a transitive process of useful information, while the Alpha Community is a process based on the initiative and popularity of some central leaders.

Contrary to anticipated results based on Granovetter’s hypothesis, in both communities studied, weak ties – or low frequency interactions, are not bridges by which new and useful information flows. That is, strong ties do not degenerate information.

**Table 9 – Exchange of useful information in the Alpha Community**

| **Model A** | **Effects** | **Parameter** | **Stderr** | **t-ratio** |  | **Effects** | **Parameter** | **Stderr** | **t-ratio** |
| --- | --- | --- | --- | --- | --- | --- | --- | --- | --- |
|  | **Markovian** | | | |  | **Markovian** | | | |
|  | **ArcA** | -2.7260 | 0.360 | 0.039* | **Model B** | **ArcA** | -2.8797 | 0.385 | 0.028* |
|  | **ReciprocityA** | 2.2394 | 0.459 | 0.052* |  | **ReciprocityA** | 2.1756 | 0.477 | 0.002* |
|  | **InStarA** | -0.0011 | 0.015 | 0.049 |  | **InStarA** | 0.0115 | 0.013 | 0.095 |
|  | **TwopathA** | 0.0373 | 0.066 | 0.047 |  | **TwopathA** | 0.0023 | 0.083 | 0.033 |
|  | **AinAoutA** | -0.9837 | 0.284 | 0.030* |  | **AinAoutA** | -0.9160 | 0.322 | -0.049* |
|  | **Social circuit** | | | |  |  | | | |
|  | **A2PA-T** | -0.0208 | 0.098 | 0.046 |  | **A2PA-T** | 0.0306 | 0.115 | 0.029 |
|  | **Categorical attribute** | | | |  | **Categorical attribute** | | | |
|  | **Realms_MatchA** | 0.7103 | 0.349 | 0.048* |  | **Realms_MatchA** | 0.8173 | 0.400 | 0.018* |
|  | **Realms_MatchRec** | 0.1514 | 0.763 | 0.057 |  | **Realms_MatchRec** | 0.0969 | 0.831 | -0.033 |
|  | **Dyadic covariate** | | | |  | **Dyadic covariate** | | | |
|  | **Low frequency**  **Weak ties** | 1.5246 | 0.228 | 0.036* |  | **High frequency**  **Strong ties** | 2.9134 | 0.318 | 0.050* |

*Significant by Wald’s test

**Table 10 – Exchange of useful information in the Beta Community**

| **Model A** | **Effects** | **Parameter** | **Stderr** | **t-ratio** |  | **Effects** | **Parameter** | **Stderr** | **t-ratio** |
| --- | --- | --- | --- | --- | --- | --- | --- | --- | --- |
|  | **Markovian** | | | |  | **Markovian** | | | |
|  | **ArcA** | -3.8280 | 0.331 | -0.038* | **Model B** | **ArcA** | -3.6550 | 0.306 | -0.028* |
|  | **ReciprocityA** | -0.3953 | 0.537 | -0.093 |  | **ReciprocityA** | -0.2404 | 0.534 | 0.064 |
|  | **InStarA** | 0.1235 | 0.033 | -0.039* |  | **InStarA** | 0.1238 | 0.031 | -0.043* |
|  | **OutStarA** | 0.1366 | 0.025 | -0.085* |  | **OutStarA** | 0.1333 | 0.025 | -0.017* |
|  | **Twopath** | -0.0539 | 0.028 | -0.044 |  | **Twopath** | -0.0707 | 0.027 | -0.011* |
|  | **Transitive-Triad** | -0.4549 | 0.208 | -0.060* |  | **Transitive-Triad** | -0.4349 | 0.209 | -0.021* |
|  | **Social circuit** | | | |  |  | | | |
|  | **ATA-T** | 1.0184 | 0.312 | -0.047* |  | **ATA-T** | 1.0647 | 0.310 | -0.019* |
|  | **Categorical attribute** | | | |  | **Categorical attribute** | | | |
|  | **Realms_MatchA** | 0.4563 | 0.257 | -0.065 |  | **Realms_MatchA** | 0.3699 | 0.252 | 0.011 |
|  | **Realms_MatchRec** | 1.5372 | 0.758 | -0.076* |  | **Realms_MatchRec** | 1.3368 | 0.769 | 0.043 |
|  | **Dyadic covariate** | | | |  | **Dyadic covariate** | | | |
|  | **High frequency**  **Strong ties** | 2.1620 | 0.206 | 0.002* |  | **Low frequency**  **Weak ties** | 1.0343 | 0.175 | -0.003* |

*Significant by Wald’s test

***Collaboration***

The third process depicts some specificities. Regarding the Alpha Community, the profile in the strength of ties is very similar. The frequency of interactions in the creation of collaborative exchange presents little difference. High frequency increases the creation of a collaborative interaction by 88.4%, while the impact of low frequency is 84.9%. Additionally, reciprocity, realms of performance and centrality are significant determinants of collaboration in that social system. In the Beta Community there is something specific; the collaborative process undergoes a transitive and semi cycle process. In this case, there is a huge difference between high and low frequencies of interactions in the creation of collaborative exchange, with 85% and 67.3% the causal effect, respectively.

**Table 11 – Collaboration in the Alpha Community**

| **Model A** | **Effects** | **Parameter** | **Stderr** | **t-ratio** |  | **Effects** | **Parameter** | **Stderr** | **t-ratio** |
| --- | --- | --- | --- | --- | --- | --- | --- | --- | --- |
|  | **Markovian** | | | |  | **Markovian** | | | |
|  | **ArcA** | -3.8928 | 0.346 | 0.038* | **Model B** | **ArcA** | -3.8262 | 0.336 | -0.006* |
|  | **ReciprocityA** | 2.4875 | 0.948 | 0.066* |  | **ReciprocityA** | 2.6325 | 0.947 | 0.069* |
|  | **InStarA** | 0.1052 | 0.076 | 0.080 |  | **InStarA** | 0.1794 | 0.074 | 0.004* |
|  | **OutStarA** | 0.1276 | 0.103 | 0.030 |  | **OutStarA** | 0.1206 | 0.100 | 0.027 |
|  | **Cyclic - Triad** | 5.2182 | 6.280 | -0.073 |  | **Cyclic - Triad** | -5.0083 | 5.466 | -0.076 |
|  | **T7A** | -0.0499 | 0.121 | 0.054 |  | **T7A** | -0.0899 | 0.110 | 0.077 |
|  | **T8A** | 0.0195 | 0.124 | 0.050 |  | **T8A** | 0.0262 | 0.127 | 0.077 |
|  | **Social circuit** | | | |  | **Social circuit** | | | |
|  | **ATA-C** | 1.7657 | 2.201 | -0.074 |  | **ATA-C** | 1.7109 | 1.937 | -0.069 |
|  | **Categorical attribute** | | | |  | **Categorical attribute** | | | |
|  | **Realms_MatchA** | 0.7672 | 0.362 | -0.005* |  | **Realms_MatchA** | 0.8624 | 0.343 | -0.025* |
|  | **Realms_MatchRec** | -1.4523 | 0.865 | -0.069 |  | **Realms_MatchRec** | -1.5282 | 0.865 | -0.006 |
|  | **Dyadic covariate** | | | |  | **Dyadic covariate** | | | |
|  | **Low frequency**  **Weak ties** | 1.7311 | 0.233 | 0.090* |  | **High frequency**  **Strong ties** | 2.0364 | 0.290 | -0.014* |

*Significant by Wald’s test

**Table 12 - Collaboration in the Beta Community**

| **Model A** | **Effects** | **Parameter** | **Stderr** | **t-ratio** |  | **Effects** | **Parameter** | **Stderr** | **t-ratio** |
| --- | --- | --- | --- | --- | --- | --- | --- | --- | --- |
|  | **Markovian** | | | |  | **Markovian** | | | |
|  | **ArcA** | -2.7585 | 0.381 | -0.021* | **Model B** | **ArcA** | -2.8218 | 0.411 | -0.014* |
|  | **ReciprocityA** | -0.5045 | 0.388 | -0.044 |  | **ReciprocityA** | -0.5876 | 0.406 | 0.039 |
|  | **InStarA** | 0.0307 | 0.026 | 0.032 |  | **InStarA** | 0.0305 | 0.028 | -0.023 |
|  | **OutStarA** | -0.0080 | 0.037 | -0.011 |  | **OutStarA** | -0.0108 | 0.036 | -0.030 |
|  | **Two Path** | -0.1091 | 0.031 | -0.047* |  | **Two Path** | -0.0998 | 0.034 | -0.016* |
|  | **Transitive Triad** | 0.0986 | 0.096 | 0.005 |  | **Transitive Triad** | 0.0858 | 0.101 | -0.011 |
|  | **Social circuit** | | | |  | **Social circuit** | | | |
|  | **ATA-T** | 0.8133 | 0.194 | -0.007* |  | **ATA-T** | 0.7812 | 0.196 | -0.016* |
|  | **ATA-C** | 0.0391 | 0.084 | -0.066 |  | **ATA-C** | 0.0315 | 0.090 | 0.011 |
|  | **Categorical attribute** | | | |  | **Categorical attribute** | | | |
|  | **Realms_MatchA** | 0.3943 | 0.215 | -0.003 |  | **Realms_MatchA** | 0.4343 | 0.233 | 0.035 |
|  | **Realms_MatchRec** | -0.7043 | 0.802 | 0.036 |  | **Realms_MatchRec** | -0.5327 | 0.878 | 0.022 |
|  | **Dyadic covariate** | | | |  | **Dyadic covariate** | | | |
|  | **Low frequency**  **Weak ties** | 0.7251 | 0.169 | -0.052* |  | **High frequency**  **Strong ties** | 1.7375 | 0.183 | 0.010* |

*Significant by Wald’s test

**Discussion and conclusion: do weak and strong ties work differently in collective action?**

On collating the former results, we can answer our research questions concisely.

|  | **Strong ties** | **Weak ties** |
| --- | --- | --- |
| **Multiplexity** | *Are strong ties determinant for the creation of multiplex ties between leaders?* | *Are weak ties determinant for the creation of multiplex ties between leaders?* |
|  | In absence of reciprocity, a high frequency of interactions increases the probability of the creation of multiplex interactions. | In a social process patterned by reciprocity, there is no substantial difference between low and high frequencies of interactions in the creation of multiplex interactions. |
|  | Among the three factors defining the strength of ties - frequency, reciprocity and intimacy - our results stress the second as a key factor that makes the difference in order to increase the probability of multiplexity. At the same time, we couldn’t ignore the fact of working in the same realm as a key factor that explains multiplex ties. | |
| **Non-multiplex or single ties** | *Are strong ties determinants for status, exchange of useful information and collaboration considered separately*? | *Are weak ties determinants for status, exchange of useful information and collaboration considered separately*? |
|  | Status: The strength of ties, whether high or low frequency, do not have a significant effect on recognizing status. We have found that the same realm of performance and reciprocity are key factors in recognizing the status of leadership.  Exchange of useful information: High frequency, reciprocal and in the same realm interactions increase the probability of exchanging useful information. In addition, transitivity is an endogenous process present in a community with a larger universe of leaders and dense interactions.  Collaboration: The pattern is similar to the exchange of useful information. The community with the smaller group of elite leaders and less dense interactions is centralized in the popularity and the initiative of few nodes. In this case, the frequency does not reveal a great difference in the increase in collaborative interactions. The larger and denser group of elite leaders depicts a transitive process, in semi-cycle, with a strong impact on frequency in the creation of collaboration. | |

Our findings reveal some facts about how social processes underpin the intersection between the strength of ties and multiplexity. In contrast with Greenbaum and Granovetter, we have highlighted that multiplexity is a function of the strength of ties. In this perspective, we have estimated how frequency and reciprocity of ties increase or not the probabilities in the formation of multiplex ties. Our discovery suggests that reciprocity and shared domains of performance fuel multiplexity, with the frequency of interactions a secondary factor. When the data are disentangled, each process reveals different associations with the three different dimensions that compose the strength of ties. Status, or the social recognition as a leader, is not a consequence of interactional frequency. Leader visibility is fostered by reciprocity and shared values in realms of social action. The exchange of useful information and collaboration are increased by the strength of strong ties considering high frequency, reciprocity, and shared realms of actions. However, high cohesive communities reveal interdependencies of social circuits – alternating transitivity – while low cohesive communities are anchored in popularity and the initiative of some leaders.

If we assume that high orders of leaders are global cohesive social circles, that is, a network of social hubs, then our findings confirm Greenbaum’s hypothesis. Multiplex ties, analyzed in entangled and disentangled ways, are strong ties determined by reciprocity, high frequency and shared domains of social action.

The findings against degeneracy of information are perhaps only an apparent rejection of Granovetter’s hypothesis. Considering the fact that we have analyzed a high social order, elite leaders in popular communities, it is easy to understand the hierarchical nature of this structure. These top-level interactional structures are cohesive clusters - the tips of social icebergs. Thus, the exchange of useful information at that top level is not necessarily a process of degeneracy, but perhaps it is the last link in a small world where information is shared.

In summary, in order to go beyond the discussion on social cohesion at local and global levels, it was necessary to dive into the social processes which could enhance multiplex and weak ties as key factors in the creation of restricted or expanded social circles. Contrary to Granovetter’s hypothesis regarding weak ties, and in syntony with Greenbaum's hypothesis about multiplexity, high order structures of leaders reveal cohesion made of strong ties defined by reciprocity, high frequency and shared realms of social action. High social orders are top levels in hierarchical social networks, so it is reasonable to think that are visible surface of interaction structures.

**References**

Babbie, Earl (1999). Métodos de Pesquisa de Survey. Belo Horizonte: Federal University of Minas Gerais Press.

Coleman, James S. (1994). Foundations of Social Theory. Cambridge, Massachusetts: The Belknap Press of Harvard University Press.

Durkheim, Émile (2014[1895]). As Regras do Método Sociológico. São Paulo: Martins Fontes.

___________ (2000[1893]). Da divisão do trabalho social. São Paulo: Martins Fontes.

Fararo, Thomas; Butts, Carter (1999). Advances in generative structuralism: Structured agency and multilevel dynamics. *The Journal of Mathematical Sociology*, 24:1, 1-65.

Gans, Herbert J. (1974). Gans on Granovetter's "Strength of Weak Ties”. *American Journal of Sociology*, 80, No. 2, Sep., 524-527.

________________ (1974). Gans reponds to Granovetter. American Journal of Sociology, 80, No. 2, Sep.,529-531.

Granovetter, Mark (1973). The Strength of Weak Ties. *American Journal of Sociology,* 78(6), p. 1360-1380.

_____________ (1974). Granovetter replies to Gans. *American Journal of Sociology*, 80, No. 2, Sep., 527-529.

______________(1985). Economic action and social structure: the problem of embeddedness. *American Journal of Sociology*, 91,481-510.

Greenbaum, Susan D. (1982) Bridging ties at the neighborhood level. *Social Networks*, 4, 367-384.

Lusher, Dean; Koskinen, Johan; Robins, Garry (2013). Exponential Random Graph Models for Social Networks. Theory, Methods, and Applications. Cambridge: Cambridge University Press.

Koskinen, Johan: Snijders, Tom (2013). Simulation, Estimation and Goodness of Fit. In: Lusher, Dean; Koskinen, Johan; Robins, Garry (2013). Exponential Random Graph Models for Social Networks. Theory, Methods, and Applications. Cambridge: Cambridge University Press.

Krackhardt, David.(1987) Cognitive social structures. *Social Networks*, 9: 109-1 3

MPNet. Wang, Peng; Robins, Garry; Pattison, Philippa (2009). Program for the simulation and estimation of exponential random graph models. Melbourne School of Psychological Sciences, The University of Melbourne.

Simmel, Georg (1999[1920]). Sociologie. Études sur les formes de la socialisation. Paris: Presses Universitaires de France.

Sampson, Robert; Raudenbush, Stephen; Earls, Felton. (1997). Neighborhoods and Violent

Crime: A Multilevel Study of Collective Efficacy. *Science*, 277, August 15th.

Sampson, Robert. (2012). Great American City: Chicago and the Enduring Neighborhood Effect. Chicago: The University of Chicago Press.

Selznick, Philip (1957), Leadership in Administration, Evanston, Ill.: Row, Peterson & Co.

_____________ (1996), Institutionalism “Old” and “`New”, Administrative Science Quarterly, 41:

270-277.

Simmel, Georg (1999). Études sur les forms de la socialization. Paris: Presses Universitaires de France.

Stivala, Alex; Koskinen, Johan; Rolls, David; Wang, Peng; Robins; Garry (2016). Snowball sampling for estimating exponential random graph models for large networks. *Social Networks*. 47,167-188.

Wellman, Barry (1979) The Community Question: The Intimate Networks of East Yorkers. *American Journal of Sociology 84 (5): 1201-1231.*

White, H., Boorman, S., Breiger, R. (1976). Social Structure from Multiple Networks I. Blockmodels of Roles and Positions. *American Journal ofSociology*, 81, 730-870.

White, Harrison (2002). Conclusion: quality is a system property. Downstream. In: Conventions and Structures in Economic Organization. Edited by Favereau, Olivier and Lazega, Emmanuel. New Horizons in Institutional and Evolutionary Economics.

________________ (2008).Identity and Control How social formations emerge**.** Second edition, New Jersey: Princeton University Press.
