# Supplementary material for: Is social cohesion produced by weak ties or by multiplex ties? Rival hypotheses regarding leader networks in urban community settings
Source: PLoS One. 2021 Sep 27;16(9):e0257527. doi: 10.1371/journal.pone.0257527 (PMC8475979; doi:10.1371/journal.pone.0257527)
Supplement: S1 Graphics — (DOCX) [file pone.0257527.s003.docx]

**Model – Community Alfa**

|  | **Estimate** | **Std. Error MCMC** | **Z value** | **Pr(>Z)** |
| --- | --- | --- | --- | --- |
| multiplex edges | -4.09958 | 0.63907 | -6.415 | < 1e-04 *** |
| edgecov.weight | 0.78201 | 0.09173 | 8.525 | < 1e-04 *** |
| isolates | -0.4803 | 0.77618 | -0.619 | 0.536044 |
| mutual | 2.45539 | 0.6883 | 3.567 | 0.000361 *** |
| mutual.cat | -2.90803 | 1.30126 | -2.235 | 0.025431 * |
| nodematch.cat | 1.13404 | 0.43908 | 2.583 | 0.009802 ** |
| gwesp.fixed.0.693 | -0.37368 | 0.35699 | -1.047 | 0.295208 |
| gwideg.fixed.0.693 | -0.82859 | 0.69373 | -1.194 | 0.232318 |
| gwodeg.fixed.0.693 | -0.88875 | 0.75741 | -1.173 | 0.240637 |

Signif. Codes : 0 ‘***’ 0.001 ‘**’ 0.01 ‘*’ 0.05 ‘.’ 0.1 ‘ ’ 1

Null Deviance: 1375.2 on 992 degrees of freedom

Residual Deviance: 240.2 on 983 degrees of freedom

AIC: 258.2 BIC: 302.3 (Smaller is better.)

**GOF-Alfa**

**
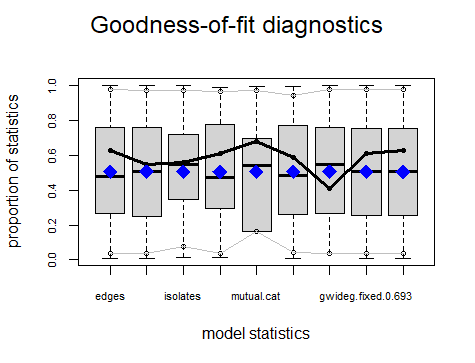
**

**
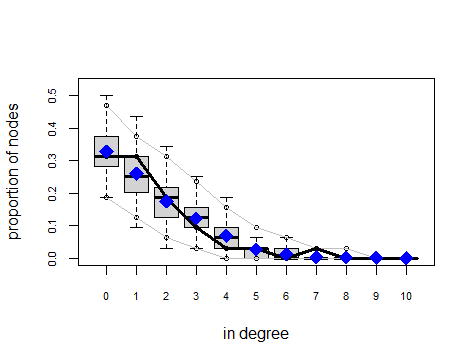
**

**
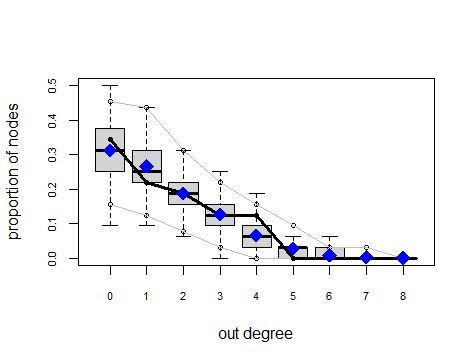
**

**
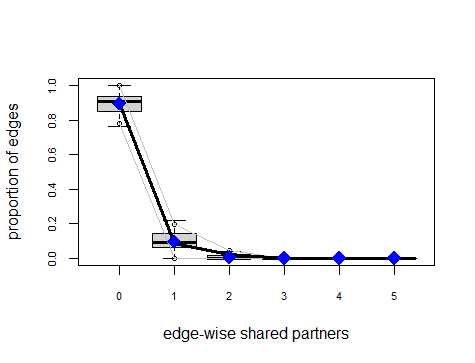
**

**
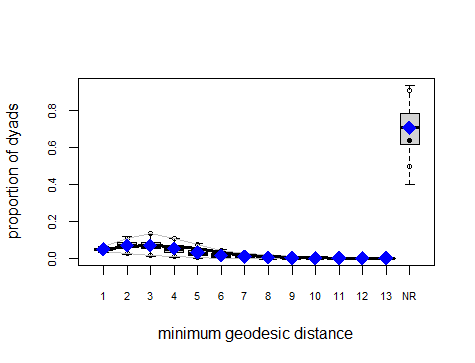
**

**Model – Community Beta**

|  | **Estimate** | **Std. Error MCMC** | **Z value** | **Pr(>Z)** |
| --- | --- | --- | --- | --- |
| multiplex edges | -5.04055 | 0.65509 | -7.694 | <1e-04 *** |
| edgecov.weight | 0.93271 | 0.09301 | 10.028 | <1e-04 *** |
| isolates | 1.75495 | 0.759 | 2.312 | 0.0208 * |
| mutual | -0.09238 | 0.77727 | -0.119 | 0.9054 |
| nodematch.cat | 0.86339 | 0.38985 | 2.215 | 0.0268 * |
| gwesp.fixed.0.693 | -0.29329 | 0.39505 | -0.742 | 0.4578 |
| gwideg.fixed.0.693 | 0.69849 | 0.76559 | 0.912 | 0.3616 |
| gwodeg.fixed.0.693 | -0.99975 | 0.63851 | -1.566 | 0.1174 |

Signif. codes: 0 ‘***’ 0.001 ‘**’ 0.01 ‘*’ 0.05 ‘.’ 0.1 ‘ ’ 1

Null Deviance: 2162.6 on 1560 degrees of freedom

Residual Deviance: 268.7 on 1552 degrees of freedom

AIC: 284.7 BIC: 327.5 (Smaller is better.)

**GOF-Beta**

**
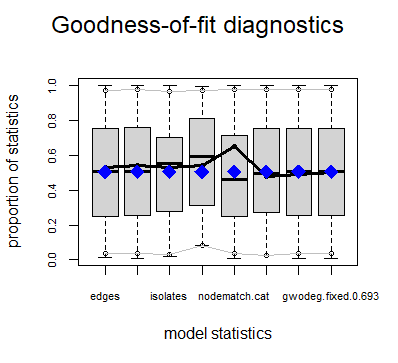
**

**
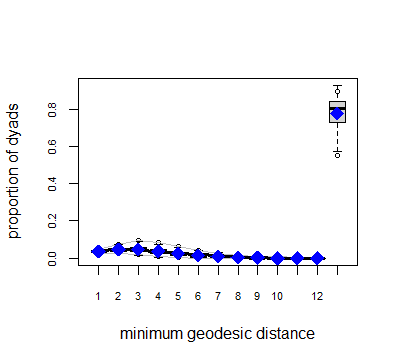
**

**
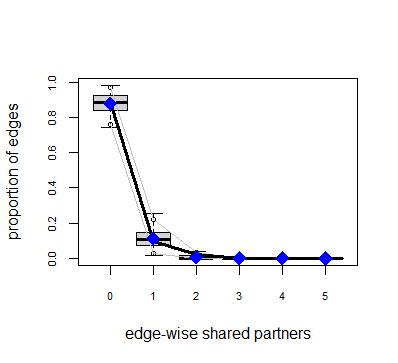
**


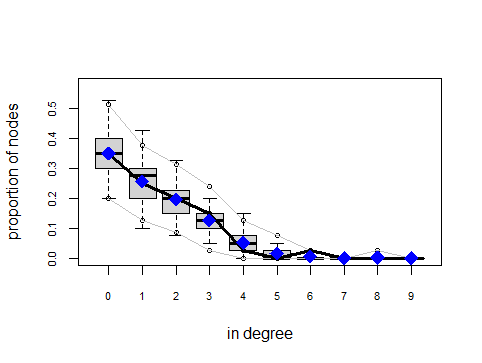
**
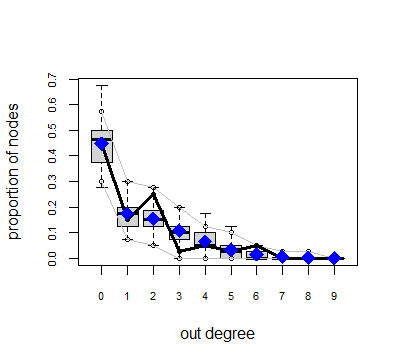
**

**
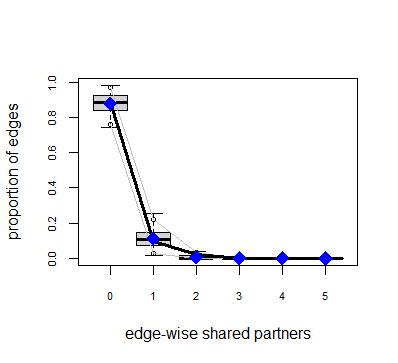
**
