# Supplementary material for: Is social cohesion produced by weak ties or by multiplex ties? Rival hypotheses regarding leader networks in urban community settings
Source: PLoS One. 2021 Sep 27;16(9):e0257527. doi: 10.1371/journal.pone.0257527 (PMC8475979; doi:10.1371/journal.pone.0257527)
Supplement: Attachment — Submitted filename: Rebbutal letter.docx [file pone.0257527.s005.docx]

Dear

Lazaros K. Gallos
Academic Editor
PLOS ONE

In the following terms, we respond the different points raised by the reviewers of our manuscript:

Is social cohesion produced by weak ties or multiplex ties?

Rival hypotheses regarding leader networks in urban community settings

In order to be clear, we have organized a table with two columns, on the left, are the original comments made the respective reviewers and on the right our positions.

| Reviewers | Our responses |
| --- | --- |
| **Reviewer 1**  “This paper aims to empirically evaluate whether cohesion is facilitated by weak ties for(sic) multiplex ties. This is an interesting question, and the authors examine it in an interesting context, however I have several concerns about the current version”: |  |
| “(1) Throughout, the writing requires proofreading to correct grammatical issues. For example, the current title is "Is made the social cohesion by weak ties or by multiplex ties?" but should be something like "Is social cohesion produced by weak ties or by multiplex ties?" | The entire manuscript was proofread by a native copyeditor, after an initial revision had been carried out by the editorial team who identified basic grammar problems, including the title. Thus, we are now certain that the mentioned error in the title has been corrected, as the reviewer and the editor can verify. Perhaps, we didn’t take enough care in some fields in the submission dashboard (metadata). In any case, the manuscript and all fields in the dashboard have now been proofread. |
| “(2) The data sharing statement is insufficient. If the authors have permission to share the data, then the data should be placed in a publicly accessible archive. If the authors do not have permission to share the data, this should be explained, and the third-party owner of the data should be identified”. | We have now included all the matrix data in the submission:   (a) Multiplex (b) Dyadic covariate (c) Categorical attribute |
| “(3) In Granovetter's original work, there was ambiguity concerning the definition of tie strength. Although Granovetter references things like interaction frequency or intensity, ultimately, he defines a tie's strength in terms of whether it is a bridge. That is, for Granovetter, a tie's strength is a structural property (what we might now call edge betweenness) and not a property of the edge itself. This ambiguity continues in this paper, where you measure the strength of ties using an interaction frequency scale. This could be a perfectly reasonable approach but is not how Granovetter conceptualized (sic) tie strength. This should be clarified”.  “(4) You operationalize tie strength using interaction frequency. However, it could also be reasonable to operationalize tie strength using multipliexity (sic): more multiplex ties are stronger. I suspect that your tie strength and multiplexity measures are highly correlated, which could cause problems for model estimation and interpretation. It would be helpful if you could report the correlation between (a) a dyad's interaction frequency and (b) their multiplexity”. | We respond to (3) and (4) together.  We agree with the fact that Granovetter defines the tie strength not only as a function of frequency and intimacy of interaction but also as structural property of weak ties. This is a consequence of his axiom: the forbidden triad. In other words, a triad composed of strong ties (high frequency and intimacy) will be a closed clique in which the shortest distance is d=1. Hence, any alternative path for overcoming the isolation of strong ties will be at least d=2 or more. In graph theory (topology), a high order network has a minimal distance between nodes d=2. This distance is the basic condition for having a graph connected beyond the dyadic order. This is why we are authorized to refer to our data as a high order of multiplex ties.  In our opinion, the forbidden triad is a seminal axiom where the intrinsic condition of ties (frequency and intimacy) and the structural bridging distance are inseparable. This position has been emphasized in the new version of the manuscript (footnote 1).  The suspicion concerning a high correlation between multiplex ties and dyadic interaction frequency has not been confirmed. The QAP dyadic correlation procedure (UCINET) has indicated a weak/moderate association of these variables for both communities.  Pearson r= 0.452 (Alpha community)  Pearson r= 0.438 (Beta community)  Regarding these results, we can say that we have not modeled a tautology.  A table with all values has been introduced in the manuscript, see page 18 |
| (5) When collecting your data, respondents were limited to 10 alters for each question. This imposes restrictions on each ego network, and on the whole network. For example, it imposes a maximum possible degree for each node, and it imposes upper limits on each of the metrics reported in Table 3. Best practice in network data collection is to allow respondents to name as many alters as they want, and not to impose such restrictions. However, because these data were collected with restrictions, they must be explicitly specified and modeled in the ERGM. | We have fitted a new model with a cutoff =10 in the nodal degree.  Nevertheless, we would like to clarify that our aim, when introducing the instruction “*You can choose up to ten names”,* was to elicit a significant number of nominees, overcoming the potential problems in the anamnesis of the respondents, rather than to limit their choices. |
| (6) The Digraphs are fuzzy and illegible. | We have included new image files. |
| (7) I had trouble understanding your models. Much of this could be clarified if you provided replication code, but in the absence of replication code: (A) Because you have data on tie strength, why not retain this information and estimate a generalized ERGM, which can use valued network data? (B) If your hypothesis concerns comparing the effect of tie strength and multiplexity, why is it necessary to consider each of the three types of ties separately? These additional analyses seem unrelated to your hypotheses and make the paper unnecessarily long. | (A)In the new version, we have modeled the data with statnet (R package). In this tool, an important number of binary ERGM statistics, particularly the ones with dyadic independence, have simple generalizations to valued ERGM. The script used is available in the new submission.  We would have liked to have modeled with the GERMG package, but unfortunately it was removed from the CRAN repository:  <https://cran.r-project.org/package=GERGM>  “Package ‘GERGM’ was removed from the CRAN repository” (Consulted on 03-05-21)”.  (B) The analyses referring to the three types separately have been excluded in order to make the paper more succinct. |
| (8) In your analysis of multiplexity in the alpha community, you write "The frequency of interactions, taken by us as a proxy of strength, reveals a difference between weak ties and strong ties." However, you do not explicitly test whether the effect of weak ties differs from the effect of strong ties. And, the observed difference between the two (92% vs. 87%) is negligible. Therefore, I do not think you can conclude that there is a difference between weak and strong ties. Although the observed difference is larger in the beta community, because there is no explicit test, you also cannot draw this conclusion there. | Because we have modeled a unique dyadic covariate with valued edges, testing for differences between weak and strong ties (after using two different and binarized dyadic networks) doesn’t make sense. |
| **Reviewer 2**  My main concern is the conceptualization of the problem. The points made by Granovetter and his cited critics are sometimes presented in a slightly different light relative to the original version, and this obfuscates the meaning and scope of the analysis |  |
| First, it should be made clear that Granovetter and his critics are all talking about large communities, where members are so numerous that there cannot possibly be strong ties linking all of them. Otherwise, the question in the title would simply not make any sense: it would be obvious that strong ties ensure cohesion. | Taking into account the hypothesis on the forbidden triad stated by Grannoveter in his seminal article “The strength of weak ties (1971)”, it is not obvious that “strong ties ensure cohesion”. This is precisely the counterintuitive sense in the Grannoveterian rationale: a social world linked by strong ties will be a fragmented world composed of clusters which are closed and disconnected. We are working exactly in this vein. Thus, we have modeled the strength of ties in order to corroborate if the cohesive interaction, in the high order, is made, or not, by weak ties. |
| Second, multiplex ties are just strong ties – whereby multiplicity of tie properties (in Greenbaum’s 1982 paper, neighborhood acquaintance and another such as kinship or shared occupation) replaces Granovetter’s measure of strength based on frequency, duration etc. So, in what respect is the opposition weak/multiplex ties new? Isn’t it just one of the many ways to assert “the strength of strong ties”, as many authors have already done since 1973? And, to go back to the first issue, aren’t multiplex ties more likely to occur in smaller communities – that is, settings different from Granovetter’s? | Clearly, when looked at in black and white, there seems to be no novelty in the opposition of weak and multiplex ties. However, what is new in our study is our attempt to test simultaneously that very opposition. Our data has been questioned for identifying if the multiplexity is matched with high or low frequency ties. In our view, there is no analytical implication, in the Kantian sense, between multiplexity and strong ties. Otherwise, to say “a tie is multiplex” does not mean that tautologically “a tie is strong”. Why presuppose that the overlap in the interaction is the same thing as high frequency? Our results indicate that multiplexity is not only a question of interactional frequency, but also an overlap of different social processes (recognition, exchange of information, collaboration) and a segmented social action restricted to domains of interest (to act to improve social services in health, education, leisure, sports, etc) separately.    The question about a greater propensity for multiplex ties in smaller communities is a challenging issue, but honestly that is not our focus and we do not have geocoded data to test it. |
| One way for the authors of this article to get around this problem could be to distinguish more explicitly between the relational dimension of social ties, however measured, and physical proximity – which would make sense in urban settings. Granovetter, Gans and Greenbaum all evoke a spatial dimension and suggest it is important, but they do not model it explicitly, so it remains fuzzy. There is a recent literature that develops on this point (eg Polge & Torre 2018, Torre et al 2019, Torre and Rallet 2005). I am not sure the authors have enough data to explore this aspect, but it would definitely be worthwhile – and in passing, it would help give a more precise definition of an “urban community”. | To distinguish between interactional proximity and spatial proximity is a good rationale for better understanding the complexity involved in cohesion. After a revision of the three proposed titles (Polge & Torre 2018, Torre et al 2019, Torre and Rallet 2005), we have included that distinction in the theoretical introduction.  However, the reality is that we do not have data to explore that distinction, because we have not collected any geographical attributes (ex. domicile, workplace) concerning the localization of leaders in the urban space of two communities. Nevertheless, we have adopted this as a strong recommendation for our future research. |
| My other concern is that there is a slight mismatch between the stated problem (ties, or absence thereof, between grassroots and the elite – which was a major point in Gans’s work, but not in Granovetter’s) and what the authors’ data are about (ties between elite members). I would invite the authors to state much more precisely how their empirical analysis operationalizes their questions. In my view, what the authors study is the relationship between frequency of interaction (the measure they take for tie strength) and shared domains of activity and performance (which is at the basis of multiplexity). But, isn’t that just a way to say that both are measures of tie strength? That they are correlated, is not so surprising: if you interact with other people in multiple social circles, then inevitably you interact with them more often, and social cohesion builds on that. The authors will recognize that I have in mind Alba and Kadushin 1976. | Let us answer in reverse order. In their classical article, Alba and Kadushin (1976) developed an algorithm to measure social proximity in terms of the overlap between structural neighborhoods. That is, assuming the same radius in the social circle, the index apprehends the proportional coincidence of alteri k for ego i and j. That measure is close to structural equivalence and other similar indexes of proximity. Without entering into technical details, it must be remembered that the key issue is to understand how the formal structure reflects the overlap in substantive social circles. In fact, Alba and Kadushin sampled an elite of intellectuals and their social exchanges in two different domains: intellectual circles and discussions on Vietnam war.  There is one similarity and one difference between the aforementioned article and our work. We have also sampled two elites of local leaders. However, our criteria for studying multiplexity are not focused on nodes and their attributes, as was Alba and Kadushin’s (inter alia, college attended, religion raised, age), who searched for the effect of node attributes on the overlap of the structural neighborhood. Indeed, we have focused on edges (the overlap of different social exchanges: recognition, information and collaboration) in order to identify the effect of strength of ties and social domain on the overlap of edges. In that vein, our results demonstrate that there would be a latent structure guided by the combinatory relations and not by the individual choice. The question posed by the reviewer has been useful in highlighting that subtle difference. What could this mean in a sociological way? In the same way as Harrison White (2008), we can say that social cohesion is something more than a formal property of a graph, as a representation of an interactional setting. It is a discipline that emerges around a social process (commit, mediate and select) and is guided by valued order (quality, prestige, and purity). We have cited White’s typology as a future path to explore the problem of community cohesion.  Finally, regarding the Granovetter vs Gangs debate and recognizing the crucial issue of having information about the links between grassroots and elite leaders, we have adopted a path similar to Robert Sampson’s strategy. Our focus was the high order (d ⪖2) of elite connections. In fact, we have trimmed the problem of cohesion targeting the boundary of social elite. Given our limited financial resources, we had two methodological options: (1) to sample probabilistically network structures of two urban sectors of Belo Horizonte (Brazil) with a high density of people (Alpha= 17.000 and Beta= 20.000); (2) to track the elite of leaders using a snowball process with key informants. We did our best when following the second option. The first option would have demanded an extremely expensive sample, taking into account state of the art sampling networks (Gjoka et al, 2014). Our focus is explicit in the title and throughout along the description of the methodology. In short, the elite connections are a key layer of social cohesion. We have included a footnote on page 10 in the new manuscript, explaining our option in the face of this methodological dilemma. |
| In the data, frequency of contact has been measured for one set of ties only, but it seems interpreted as strength of ties overall. Perhaps results should be interpreted more cautiously (for example, someone named for collaboration but not for status may be a very frequent contact). | The frequency of contact was measured, in a general sense (*how often have you spoken with each of them*) and once, with the first question, because the respondents refused to answer twice or more, explaining that they had already answered that issue. In fact, to decompose the anamnesis for three different issues was not easy and caused irritation. |
| PP. 9 and 13: Granovetter did write “In the absence of actual network data, all this is speculation” but he was referring specifically to the study of Gans, who hadn’t collected such data. He wasn’t suggesting that it is always challenging to sample a structured set of interactions. By the way, I am not sure what the authors mean when they refer to the problem of non-independence of observations in network data, p. 13. The problem is not to eliminate dependence, but to control or (better) to model it, as ERGM do | On page 3 we have cited Grannoveter:  “(…) Nor has comparable information been collected in any context”. Obviously, he wrote this in 1973. In fact, the statistical research on network sampling is working on robust and succinct solutions to that inferential problem.  We agree with the reviewer. To prevent any misunderstanding, we have erased the ambiguous phrase: “Therefore, the challenge consists in preventing one observation from introducing selection bias into others”. |
| P. 14, especially Table 1: what is meant precisely by “global” and “local”? | Global refers to spatially sparse networks and local to spatially close networks (see page 8 in the manuscript). |
| P. 15 The authors mention data on negative ties (conflict etc.) and bridging social capital without giving any details. But because they do not use these data, I suggest removing the mention at all, and focusing only on the data used in the analysis. | Good tip. We have removed any mention of other data. |
| P. 19 The two groups of elite members are pretty small (32 and 40 people respectively). But how large are the communities of which they are the elites? This is important to understand the extent to which Granovetter’s framework applies (my comment above). | Above, we have declared the seize of both urban sectors. |
| P. 24, What is the difference between the left and right columns of Table 5? The effects are the same, but parameter values, standard errors and t-ratios are not. Why are the effects of the models for Beta different from those of Alpha? What is the significance level chosen – 5%? There is a typo in the Reciprocity effect of the left column: the t-ratio should not be negative. | We have plotted new models for both communities, including p values. This time, and after inspection on parameter values, there is no difference between theta coefficients of edge valued covariates (t test for two samples). So, we did not point out that fact. |
| PP. 24-25, The comments do not match the figures in the table. For example, the Strong Ties coefficient of Model B for community Alpha is 3.1819, but it is said that “strong ties […] increase the probability of forming multiplex links by 92%”: why? If any transformations are required to make the table values interpretable, they should be stated clearly. | In the new tables there are no not such problems because we have included a unique covariate for the strength of ties. |

Gjoka, M., Smith, E., and Butts, C. (2014). Estimating clique composition and	size distributions from sampled network data. In 2014 IEEE Conference on	Computer Communications Workshops (INFOCOM WKSHPS), pages 837-842.

Sincerely

Silvio Salej Higgins
