# Supplementary material for: Is social cohesion produced by weak ties or by multiplex ties? Rival hypotheses regarding leader networks in urban community settings
Source: PLoS One. 2021 Sep 27;16(9):e0257527. doi: 10.1371/journal.pone.0257527 (PMC8475979; doi:10.1371/journal.pone.0257527)
Supplement: Attachment — Submitted filename: Response to Reviewers 2.docx [file pone.0257527.s006.docx]

PONE-D-20-24819R1
Is social cohesion produced by weak ties or by multiplex ties?
Rival hypotheses regarding leader networks in urban community settings
PLOS ONE

Dear

Lazaros K. Gallos
Academic Editor
PLOS ONE

After a careful reading, we respond the valuable comments and recommendations made by the reviewer in the second round. The editorial feedback has been constructive and has permitted enhance the quality of our work.

In order to be clear, we have organized a table with two columns, on the left, are the original comments made the respective reviewers and on the right our positions.

| Reviewer | Our responses |
| --- | --- |
| “The authors have done a good job addressing most of my original comments, however one of my more overarching original concerns remains. In my first review, I commented that multiplexity and frequency could both be viewed as measures of tie "strength." This is a point raised by Reviewer #2 also, and indeed Granovetter (1973) himself wrote that "most multiplex ties [are] strong" (p. 1361). Several features of the current paper confirm this point. For example, on page 5 you summarize Our findings reveal some facts about how social processes sustain cohesion in the high-order structure of elite connections. These results suggest a segmented multiplexity in specialized domains of performance that is fueled by both a high frequency of interactions and reciprocal relationships. Greenbaum's finding by mentioning "strong multiplex ties" and on page 17 you find that frequency and multiplexity are highly correlated at r > 0.4 (despite your claim to the contrary, I view this as a large correlation)”. | Definitively, we have been persuaded by the reviewer’s overarching concern regarding the dual aspect of strength: multiplexity and frequency of ties.  On page 12 we have included Footnote #5 citing Granovetter’s seminal insight about the dual aspect of strength: frequency of ties and multiplexity.  On page 17, and following the general focus recommended by the reviewer (see below), we have modified our interpretation about correlations between multiplexity and frequency: “The Pearson coefficients of 0.452 and 0.438 can be considered as positive high correlations between multiplex ties and weekly interaction. In other words, multiplexity and frequency are associated as indicators of the strength of ties. |
| “For these reasons, I continue to believe that multiplexity and frequency are both measuring different aspects of a common construct called "strength". This raises at least two issues:  First, because others have previously used both frequency and multipliexity as indicators of strength, it is confusing that you treat frequency as an indicator of strength, but not multiplexity. It would be clearer if you identified both as indicators of strength, and throughout the paper simply examined the relationship between frequency and multiplexity”. | Taking into account this valuable recommendation, we have carefully revised the entire manuscript and introduced changes (highlighted in green in the track version) aiming to maintain this general rationale.  P.1 The second half of the abstract has been changed.  P. 9, Table 1 - we have changed the column titles.  P.12, Table 2 - we have introduced “frequency of ties”  P.12 This new phrase precedes Footnote #5 “The scale of frequency in the first sociometric generator, when added to the multiplexity of networks, enabled us to operationalize a common construct for the strength of ties as can be inferred from Granovetter’s (1973) seminal work”.  P.17, Table 4, we have changed the column title and rewritten the research question: “*How do the strength of ties (multiplexity and frequency) and other endogenous/exogenous factors determine the emergence of higher-order structure of elite connections”*  P.17 In order to maintain the coherence between the research question and theoretical framework, we have introduced:  “In an attempt to answer the former question, we modeled the higher-order structure of elite connections (…) We asked if the network of leaders is a combinatory result of the strength of ties, measured by multiplexity and frequency, realms of action and some patterns of interaction”.  “As a preliminary model, we tested the linear correlation between the multiplex ties and the dyadic covariate strength of ties. In Table 5, the QAP dyadic correlation procedure indicated a clear association of variables for both communities”:  P.17 We have included the footnote #6. |
| Second, your finding that "the strength of ties [i.e. frequency] has a positive effect on multiplexity" seems obvious given prior work that has treated frequency and multiplexity as both indicators of strength, and given your own observed correlation between the two. You go on to claim that this finding is not trivial and that "there is something subtle here." Perhaps, but it is so subtle that I was unable to understand why this finding is not trivial. If the core finding of your analysis is that the association between frequency and multiplexity is *not* trivial, the reasons why it is not trivial need to be clearer. | We have introduced some changes in the presentation of results. In the tables of models, we have detected some minor errors with points of thousands in some estimates, which was because the original output in R was a txt file that was then copied via windows excel. Now, all the data are exactly equal to the model outputs.  P. 18 “Tables 6 and 7 depict the high order structure of elite connections for both communities. Each model includes two kinds of effects: endogenous – multiplex edges, isolates, mutual, edge covariate with frequency - and exogenous – categorical attribute with the realms of interaction”  P. 19, Tables 6 and 7, we have introduced “multiplex edges” because this is clearer regarding the dual aspect of strength.  P.19 and 20. We were taking not in terms of triviality but in terms of expected results. In this way, on the one hand, we recognize that the statistical association between multiplexity and frequency of ties is aligned with previous studies, and on the other, we focus the novelty of our results in terms of a segmented multiplexity which doesn't correspond to overlapping social circles.  “From the point of view of Granovetter’s definition of strength and Greenbaum’s results, this result is expected. Nevertheless, there is something new in these communities: multiplexity is not simply the overlap of social circles. Differently from the standard concept of multiplexity, the conjunction of three different processes (status, exchange of information and collaboration) occurs in a segmented way, in clearly differentiated, that is specialized, realms (education, religion, politics, security, health, business, local organization, sports, and leisure), where the leaders exercise their authority and participate in mediation processes.  Discussion and conclusion  “Our findings reveal some facts about how social processes sustain cohesion in the high-order structure of elite connections. These results suggest a segmented multiplexity in specialized domains of performance that is fueled by both a high frequency of interactions and reciprocal relationships.” |
| As a more minor, but still important, point - Your analyses hinges upon results from two ERGM, however I did not see any evidence of these models' goodness-of-fit. This should be included to confirm that the model estimates are reasonable. | This was indeed a was regrettable omission. This time we have included the GOF of the two models. |

With our best wishes,

Silvio Salej Higgins (main author)

16/08/2021
